# Impact of Clinical Information on the Turnaround Time in Surgical Histopathology: A Retrospective Study

**DOI:** 10.7759/cureus.2596

**Published:** 2018-05-08

**Authors:** Syed Muhammad Hammad Ali, Usama Muhammad Kathia, Muhammad Umar Masood Gondal, Ahsan Zil-E-Ali, Haseeb Khan, Sabiha Riaz

**Affiliations:** 1 Pharmacology, Fatima Memorial Hospital College of Medicine & Dentistry, Lahore, PAK; 2 Department of Surgery, Fatima Memorial Hospital, Lahore, PAK; 3 Medical Student, Fatima Memorial Hospital College of Medicine & Dentistry, Lahore, PAK; 4 General Surgery, Fatima Memorial Hospital, Lahore, PAK; 5 Department of Pathology, Fatima Memorial Hospital College of Medicine & Dentistry, Lahore, PAK

**Keywords:** histopathology, surgical pathology, turnaround time, clinical audit, clinical information, quality improvement

## Abstract

Introduction

Clinical information (CI) is a key requisite to diagnose and report a specimen in histopathology. A timely dispatched report can help a clinician to confirm a diagnosis and initiate a prompt treatment plan while an unnecessary delay in reporting time can compromise patient's healthcare. The aim of this study was to ascertain the impact of the adequacy of CI provided by clinicians on the turnaround time (TAT) and to investigate factors pertinent to specimens, their handling, and diagnosis.

Methods

This retrospective study reviewed a total of 803 surgical specimens reported in a duration of four months (from December 2015 till March 2016) by the Department of Histopathology, FMH College of Medicine & Dentistry, Lahore, Pakistan. Frozen section and cytology specimens were excluded. CI was classified into three categories: short and focused, long and detailed, and deficient CI. Deficient CI was designated where the pathologist had to seek more information from the requesting clinicians. Total time taken by the histopathologist to complete a report was calculated after excluding weekends and holidays. Other factors like type of specimen, special staining, diagnosis of malignancy and source of referral were also studied. The data were entered and analyzed on SPSS 22.0 (IBM, Armonk, NY). Shapiro-Wilk test was used to measure the distribution.

Results

Most of the specimens (46.2%, n = 371) were reported within three days. Of these, most of the specimens (46.9%, n = 174) had a short and focused CI (p < 0.001). Majority of the specimens which were reported within four to five days (42.1%, n = 114) and after five days (62.1%, n = 100) were found to have a long and detailed CI in their requisition forms. Median TAT extended to six (4.00-7.00) days with the use of special stains (p < 0.001). One hundred and sixty-three (20.29%) of the total cases were diagnosed as malignant in which the median TAT significantly prolonged to five days (p < 0.001). Most of the specimens (80%, n = 60) received from the outside laboratories had a long and detailed CI in requisition forms. Endometrial tissue specimen was the predominant type received by the department (24.3%, n = 90).

Conclusion

Adequate CI is necessary for timely and error-free reporting of a specimen in surgical histopathology. A short, focused and concise CI is associated with a shorter TAT. Long and detailed CI is often seen with a complex surgical specimen that requires a longer time to report. Factors like specimen type, special staining, number of special stains and diagnosis of a malignancy also affect TAT.

## Introduction

Histopathology is one of the rapidly evolving specialties that are being considered as the cornerstone of modern medical sciences. Almost every allied branch of medicine and surgery is somehow dependent upon a histopathologist for establishing of a definitive diagnosis. The decisive nature of histopathology reporting thus necessitates maintenance of a standard and quality in reporting.

By quality, we mean a timely done, well-elaborated reporting of the specimen with the utmost diagnostic accuracy [1]. Out of several factors determining the quality of reporting in histopathology, turnaround time (TAT) or timeliness is a key factor from both the clinician’s and patient’s perspective. A timely reported specimen with the most accurate diagnosis can help the clinician to devise a prompt and definitive treatment plan. Also, a delay in reporting time can significantly compromise the quality of healthcare and increase mortality [2].

While histopathologists are expected to help clinicians make a diagnosis in the most accurate way, it is on the part of clinicians to provide them with adequate and pertinent clinical information [3]. Without a deep insight into the patient’s clinical information, provisional diagnosis and purpose of the biopsy, a histopathologist is unable to see the specimen from clinician’s point of view. Therefore, the histopathology report might fail to address the desired queries and fall short of the clinician’s expectation. Also, an inadequacy of the provided clinical information may cause a delay in reporting time and may lead to inaccurate diagnosis [4].

The focus of this study was to determine whether the provision of clinical information (CI) along with the surgical histopathology and biopsy specimens has an impact on the TAT besides other laboratory and specimen factors, in an academic institution.

## Materials and methods

A retrospective observational study was conducted in the Department of Histopathology, FMH College of Medicine & Dentistry, Lahore. The surgical histopathology specimens and biopsies reported from December 2015 till March 2016 were reviewed after getting ethical approval from the institutional review board (IRB).

Any specimen received from a patient during a major or minor surgical procedure was termed as a *surgical specimen*. Those specimens, requiring hematoxylin & eosin (H&E) staining only, were termed as a *routine specimen*. Total time taken by the histopathologist to report a specimen from the time of its receipt in the lab was defined as the turnaround time (TAT). This included the specimen processing, gross sectioning, slide preparation, fixation, routine and special staining. Gazetted holidays and weekends were excluded.

Specimens were classified into three groups on the basis of the CI provided in their requisition forms. These groups were named as: Long and detailed, short and focused, and deficient CI (Table 1). The entire surgical pathology and biopsy specimens received and reported during these four months were included. Cytology and frozen section specimens were excluded.

**Table 1:** Grouping of the specimens based on the adequacy of clinical information (CI) provided by the clinician in the requisition forms.

Clinical Information (CI) Groups	Operational Definitions
Long & detailed	Complete CI including patient’s past clinical history and/or co-morbidities, type of procedure done, site of biopsy, intraoperative and postoperative findings and provisional diagnosis.
Short & focused	One or more of the above criteria were missing but the CI was sufficient enough for the pathologist to make a diagnosis and complete the report.
Deficient	Absence of CI or (if provided) some of the above criteria was missing. The pathologists had to seek more CI from the requesting clinician/lab to establish a diagnosis and complete the report.

Data was collected and categorized using a specially designed tool. Following variables were observed for each of the specimens: the specimen type, adequacy of provided CI, TAT (in days), special staining (Immunohistochemical, histochemical stains, and immunofluorescence), number of special stains used, diagnosis of malignancy and source of referral. Data was then entered and analyzed using SPSS 22.0 (IBM, Armonk, NY). Shapiro-Wilk test was used to determine the distribution of data. Mean ± SD is given for normally distributed variables and median (IQR) is given for non-normally distributed variables. For the purpose of comparison, One-way ANOVA was used for normally distributed data while Kruskal-Wallis test and Mann-Whitney U-test were used for the non-normally distributed data. Chi-square test of significance was used to measure associations. Spearman correlation was used to study the correlation between ordinal and quantitative variable. For all purposes p < 0.05 was considered statistically significant.

## Results

A total of 803 specimens were reported in a period of four months. The median TAT for all specimens was four days (3.00-5.00). Highest reported specimens were the breast tissue (18.2%, n = 146), followed by the endometrial (16.3%, n = 131) and gastrointestinal (GI) biopsies (12.6%, n = 101).

Majority of the specimens (46.2%, n = 371) were reported within three days, while another 271 (33.8%) of the total specimens were reported within five days of receipt. Most of the specimens reported within three days were endometrial (24.3%, n = 90) and GI biopsies (16.4%, n = 61). While most of the specimens reported after five days were the renal biopsy (36.0%, n = 58) and breast tissue specimen (22.4%, n = 36). Longest median TAT was seen with liver and renal biopsy specimens, that is, 7.50 days and 7.00 days (5.00-10.00), respectively. Thyroid tissue specimens had the shortest median TAT of two days (2.00-3.00). A significant association was seen between groups based on the adequacy of CI and the categories of TAT (p < 0.001). Most of the specimens reported within three days (46.9%, n = 174) were found to have a short and focused CI. Majority of the specimens reported within four to five days (42.1%, n = 114) and more than five days (62.1%, n = 100) had a long and detailed CI in their requisition forms (Table 2).

**Table 2:** Contingency table showing significant association between adequacy of clinical information (CI) and turnaround time (TAT).

	Turnaround Time (TAT)			Total n (%)	p-value
	3 days	4-5 days	More than 5 days		
Clinical Information (CI)	n (%)	n (%)	n (%)		
Long & detailed	123 (33.2)	114 (42.1)	100 (62.1)	337 (42.0)	< 0.001
Short & focused	174 (46.9)	103 (38.0)	28 (17.4)	305 (38.0)	
Deficient	74 (19.9)	54 (19.9)	33 (20.5)	161 (20.0)	
Total n (%)	371 (100)	271 (100)	161 (100)	N = 803	

Special stains were performed on 213 (26.5%) of the total specimens. Median TAT for the specimen with special stains, 6.00 (4.00-7.00) days, was longer as compared to the TAT for specimen without special stains, 3.00 (2.00-4.00) days (p < 0.001). Out of these 213 specimens, median TAT was 6.00 (4.00-8.50) days in long and detailed CI group (66.1%, n = 141), 4.00 (4.00-6.50) days in short and focused CI group (11.7%, n = 25) and 5.00 (4.00-7.00) days in deficient CI group (22.0%, n = 47).

Among the specimens with special staining, 102 (47.9%) were the breast tissue and 74 (34.7%) were the renal biopsy specimens. The median number of special stains used on renal biopsy specimens (n = 5.00) was significantly higher as compared to breast tissue (n = 3.00) and the remaining specimens (n = 3.00) (p < 0.001).

Out of the entire surgical specimens, 163 (20.29%) cases were diagnosed as malignant. The highest number of malignancy was reported in the breast tissue specimens (73.0%, n = 119) followed by the GI biopsies (4.9%, n = 08). Median TAT was significantly longer, that is, 5.00 days (3.00-6.00) for specimens with a diagnosis of malignancy in contrast to only 3.00 days (3.00-5.00) for specimens without a malignancy. Finally, a significant association was seen between the CI groups and the categories based on the source of referral. Majority of the specimens (80%, n = 60) referred from the outside labs had a long and detailed CI (p < 0.001).

## Discussion

TAT is important when we assess the quality of reporting in histopathology. It is a key parameter that is critically viewed by the clinicians and patients to ascertain the performance of any lab. TAT is also related to lowest satisfaction rates amongst the clinicians as a majority of them are not satisfied with the reporting time [5].

Ferrara et al. concluded in their study on malignant skin lesions that providing pertinent CI to the histopathologist can actually reverse their initial diagnosis [6]. Therefore, insufficient clinical information on the requisition form can often mislead the histopathologist causing an unnecessary delay in reporting time [7]. Yet there is a paucity of data relating adequacy of provided CI with the TAT. There are no clear-cut guidelines in the literature as to ‘how much’ of a CI is required by a histopathologist to report a surgical specimen without causing delay. Therefore, we came up with our own definitions of CI groups, to assess the impact of provided CI on TAT.

In our study, a majority of the specimens (57.0%) within short and focused CI group were the ones to be reported within three days. While it took more than three days to report most of the specimen (63.5%) within long and detailed CI group. There may be several explanations for these paradoxical findings. First of all, most of the times a short but focused CI is provided by the physician with simple cases, like cholelithiasis in case of gallbladder specimen. While more complex the case is, the physician tends to provide long and detailed CI addressing every aspect of the clinical case. Such type of CI is frequently seen in case of renal biopsies, lymph nodes and soft tissue specimen, where the clinician is uncertain about the medical diagnosis of the patient. However, this is not always the case as we do come across complex specimens with insufficient CI that falls short of pathologist’s requirements. Other factors like the specimen type, special staining, numbers of special stains used and diagnosis of a malignancy may have a confounding effect on TAT.

In our study, 371 (46.2%) of the entire specimens were reported within three days. In a developing country like Pakistan, this is a fairly acceptable mark. The College of American Pathologists (CAP) recommended a TAT of two days for reporting of most of the routine specimens [8]. However, it was then removed from their checklist in 2011 (ANP.12150). Our departmental policy is to report most of the routine specimens (those requiring H&E staining only) within two days or to generate and deliver a complete report within three days.

Coard and Gibson reported a TAT of three days, for 66% and six days, for a cumulative 89% surgical pathology specimen in a hospital in West Indies [9]. In our study, 87% of the specimens were reported within six days. Patel et al. reported a TAT of two days, for 77% of their entire 713 non-biopsy surgical specimens. In their study, prolonged TAT was associated with immunohistochemical (IHC) staining, diagnosis of a malignant disease, having the opinion of other pathologists and frozen sections [10].

Chan et al. reported a mean TAT of 11.55 ± 11.38 days for 749 oral histopathology specimens, in a lab of Malaysia, which was longer than our median TAT of four days. In their study, TAT was largely affected by the specimen types however special stains and IHC stains did not influence the TAT [11].

In general, the breast tissue, renal biopsy, and lymph node specimens require the use of special stains. In case of a breast tissue specimen, if a malignancy is diagnosed, several prognostic markers including ER, PR, and HER2/neu are performed. Likewise, at least five immunofluorescence stains are performed on renal biopsy specimens, in routine, to establish a medical diagnosis in our settings. Special stains are also used on lymph node specimens to rule out various lymphoproliferative disorders. A delay in reporting time is often observed for the specimens with special staining. In our study, the median TAT dropped to three days after excluding the breast and renal biopsy specimen alone. The median TAT for all renal biopsy specimens (n = 78) was seven days (5.00-10.00), even though 88.5% of these specimen were within long & detailed CI group. However, special staining was done on 100% of these specimens. Similarly, special staining was performed on 78.6% of the breast tissue specimens with long and detailed CI. The use of additional stains in a majority of these specimens might account for the longer TAT despite complete CI.

TAT varied significantly with the adequacy of provided clinical information in case of the breast tissue, soft tissue and skin biopsy specimens (Table 3). Breast tissue specimen within deficient CI group took a long time to report. Most of the soft tissue specimens are tricky to diagnose and require additional stains to determine their embryonic origin and tissue type. Moreover, sarcomas are frequently encountered in our settings that require more careful and elaborated reporting that may prolong TAT. Cytogenetic analyses are routinely performed to subtype soft tissue sarcomas worldwide [12]. However, lack of these cytogenetic analyses in our settings further delays reporting time along with additional use of special staining.

**Table 3:** Comparison of turnaround time (TAT) of different surgical histopathology specimen types on the basis of clinical information (CI). BSO: Bilateral salpingo-oophorectomy; GI: Gastrointestinal; ENT: Ear Nose Throat; TAT: Turnaround time. ^a^**Values are given as mean ± SD for normally distributed variables and median (IQR) for non-normally distributed variables. ^b^p-value not calculated due to the limitation of the statistical test. ^c^TAT was constant. ^d^**p-value is generated by Kruskal-Wallis test. ^e^p-value is generated by One-way ANOVA. ^†^Unclassified specimens like a scrotal cyst, subcutaneous cyst, perianal fistulas, pancreas, and spleen etc. ** *p-value < 0.05 is considered statistically significant.

		Clinical Information (CI)						
		Long & detailed		Short & focused		Deficient		
	Specimen Type	n (%)	TAT (days)^a^	n (%)	TAT (days)^a^	n (%)	TAT (days)^a^	p-value
1	Maxillofacial & Oral (including salivary glands)	16 (4.7)	4.06 ± 1.38	0 (0.0)	-	1 (0.6)	4^ c^	-^b^
2	Renal Biopsy	69 (20.5)	7.00 (6.00-11.00)	7 (2.3)	6.42 ± 2.22	2 (1.2)	6.00 ± 2.82	0.525 ^d^
3	Breast Tissue	61 (18.1)	4.00 (3.50-5.00)	27 (8.9)	3.00 (2.00-4.00)	58 (36.0)	5.00 (3.00-6.00)	0.017 ^d^*
4	Endometrium	47 (13.9)	3.00 (2.00-4.00)	59 (19.3)	3.00 (2.00-4.00)	25 (15.5)	3.00 (2.00-4.00)	0.49^d^
5	Uterus	7 (2.1)	3.00 (3.00-5.00)	19 (6.2)	3.00 (2.00-4.00)	4 (2.5)	3.25 ± 1.25	0.603^d^
6	Uterus with ovaries (BSO)	11 (3.3)	4.00 (3.00-6.00)	12 (3.9)	3.83 ± 1.26	11 (6.8)	4.18 ± 1.99	0.741^d^
7	Fallopian Tubes	5 (1.5)	3.20 ± 0.83	10 (3.3)	3.00 ± 1.05	9 (5.6)	3.22 ± 1.09	0.882^ e^
8	Soft Tissue	13 (3.8)	6.69 ± 3.40	9 (2.9)	4.00 ± 1.73	5 (3.1)	3.00 (2.00-5.00)	0.028^ ​​​​​​^^d^*
9	Thyroid Tissue	2 (0.6)	2^ c^	1 (0.3)	2^ c^	4 (2.5)	3.00 ± 1.41	0.605^ e^
10	Lymph Node	8 (2.3)	4.75 ± 1.98	5 (1.6)	4.20 ± 1.64	3 (1.8)	3.00 ± 1.00	0.368^ e^
11	Liver Biopsy	1 (0.3)	6^ c^	0 (0.0)	-	1 (0.6)	9^ c^	-^b^
12	Gall Bladder	13 (3.9)	3.00 (3.00-4.00)	26 (8.5)	3.00 (2.00-4.00)	2 (1.2)	2^ c^	0.18^d^
13	Upper and Lower GI	10 (3.0)	3.90 ± 1.52	21 (6.9)	3.00 (2.00-4.00)	6 (3.7)	4.50 ± 2.58	0.546^d^
14	Male Reproductive/ Urinary Tract	3 (0.9)	6.00 ± 4.35	5 (1.6)	3.20 ± 0.83	1 (0.6)	6^ c^	0.344^ e^
15	GI Biopsy (endoscopy/colonoscopy)	30 (8.9)	3.00 (2.00-5.00)	63 (20.7)	3.00 (2.00-4.00)	8 (5.0)	4.00 (3.00-4.75)	0.201^d^
16	Musculoskeletal (muscle, bone)	11 (3.3)	5.27 ± 2.05	4 (1.3)	4.00 (3.25-4.00)	1 (0.6)	6^ c^	0.168^d^
17	Ovarian Cyst	5 (1.5)	4.20 ± 2.48	6 (2.0)	3.66 ± 1.50	3 (1.9)	3.33 ± 1.15	0.805^ e^
18	ENT (tonsils, polyps, etc.)	2 (0.6)	3^ c^	5 (1.6)	4.40 ± 1.34	1 (1.6)	5^ c^	0.357^ e^
19	Neurosurgery (nerve, brain biopsy, etc.)	1 (0.3)	3^ c^	1 (0.3)	6 ^c^	0 (0.0)	-	-^b^
20	Skin Biopsy	1 (0.3)	9 ^c^	3 (1.0)	4.34 ± 1.15	3 (1.9)	3.67 ± 1.15	0.038^ e^*
21	Others ^†^	21 (6.2)	4.28 ± 1.52	22 (7.2)	3.00 (2.00-4.00)	13 (8.1)	3.61 ± 0.96	0.157 ^d^
	Total (N = 803)	337 (42.0)		305 (38.0)		161 (20.0)		

Volmar et al. reported a median TAT of 2.72 days, for 2763 large or complex specimen and found malignancy, governmental institutions, special handling, frozen section and mandatory overnight fixation as the main factors associated with prolonged TAT [13].

In our study, diagnosis of malignancy had a significant association with prolonged TAT. It is related to the fact that diagnosing a malignancy frequently requires detailed examination, resection margin assessment, grading and staging workup besides intradepartmental consultation. In our settings, the histopathology slides are reviewed by multiple pathologists before labeling it as malignant and for further subtyping. Additional sections from gross specimens are drawn and additional staining is also performed in such cases, whenever required.

Majority of the specimens with long & detailed CI were referred from the outside labs. It suggests some clumsiness on part of the referring doctors from our own teaching hospital. In our settings, the junior house officers are sometimes responsible for filling up the requisition forms and handing them over to the patients or their attendants. Unfortunately, they are not trained in this regard and are on their own for most of the times. However, obtaining CI from inpatient departments of our teaching hospital is easy and effortless. In the routine, our pathologists, after receiving such specimens with deficient CI, make efforts to contact and acquire CI from the referring clinician as soon as possible.

Nakhleh and Zarbo reported absent clinical information in 2.4% of the histopathology specimen, from 417 labs of the United States [14]. However, in our study, deficient CI was seen in 161 (20.0%) of the entire specimens, which are lower than 170 (34.0%) of the total 500 specimens reviewed in a lab of Pakistan by Sharif et al. [15]. The lower percentage in our study might be due to the fact that majority of the cases we reviewed were referred from our own teaching hospital.

In another study, improved TAT was seen in non-teaching institutions that did not have any pathology training program [16]. Our study was conducted in a single institution with undergraduate and postgraduate teaching programs. In our settings, the resident doctors examine and review the slides along with a consultant pathologist at the same time. Therefore, the training program has no impact on TAT of our histopathology department.

There were few limitations to our study. We did not study the effect of simple and complex surgical specimens as the latter ones require extensive processing than the simple surgical specimens. Furthermore, we did not break up TAT into its individual components like the accessioning of the specimen, the intralaboratory phase including overnight fixation and processing of the specimen and then the time taken outside of the lab, that is, the typing and delivery of the report. However, both the clinicians and patients are affected by the overall TAT, studying individual components of TAT can be helpful for departmental purposes only. We did not study the effect of decalcification of specimens on TAT as bone specimens are rarely received at our lab.

## Conclusions

We conclude from our study that, even though an adequate CI is necessary for a pathologist to appropriately address the queries of a requesting clinician, it does not significantly improve the TAT. Routine cases are the ones to get reported early and in such cases a short but focused CI is usually enough to report the specimen. However, a detailed CI is often required by the pathologist to report complex cases that require special handling and have longer TAT. Nonetheless, clinicians are the ones to see the patients in person and thoroughly assess them. They should also facilitate the pathologists by providing them with a complete and focused clinical picture in the requisition forms. Associated factors like the diagnosis of a malignant disease, IHC staining, numbers of special stains and specimen type can also influence the TAT.
